# CD8^+^ T cell exhaustion and its regulatory mechanisms in the tumor microenvironment: key to the success of immunotherapy

**DOI:** 10.3389/fimmu.2024.1476904

**Published:** 2024-09-20

**Authors:** Biao Zhang, Jinming Liu, Yuying Mo, Kexin Zhang, Bingqian Huang, Dong Shang

**Affiliations:** ^1^ Department of General Surgery, Clinical Laboratory of Integrative Medicine, The First Affiliated Hospital of Dalian Medical University, Dalian, China; ^2^ Department of Oncology, First Affiliated Hospital of Dalian Medical University, Dalian, China; ^3^ Central Laboratory, The First Affiliated Hospital of Dalian Medical University, Dalian, China; ^4^ Key Laboratory of Clinical Cancer Pharmacology and Toxicology Research of Zhejiang Province, Department of Clinical Pharmacy, Affiliated Hangzhou First People’s Hospital, Westlake University, Hangzhou, China; ^5^ Institute (College) of Integrative Medicine, Dalian Medical University, Dalian, China

**Keywords:** CD8 + T cell, T cell exhaustion, tumor microenvironment, immune checkpoint blockade, adoptive T cell treatment

## Abstract

A steady dysfunctional state caused by chronic antigen stimulation in the tumor microenvironment (TME) is known as CD8^+^ T cell exhaustion. Exhausted-like CD8^+^ T cells (CD8^+^ Tex) displayed decreased effector and proliferative capabilities, elevated co-inhibitory receptor generation, decreased cytotoxicity, and changes in metabolism and transcription. TME induces T cell exhaustion through long-term antigen stimulation, upregulation of immune checkpoints, recruitment of immunosuppressive cells, and secretion of immunosuppressive cytokines. CD8^+^ Tex may be both the reflection of cancer progression and the reason for poor cancer control. The successful outcome of the current cancer immunotherapies, which include immune checkpoint blockade and adoptive cell treatment, depends on CD8^+^ Tex. In this review, we are interested in the intercellular signaling network of immune cells interacting with CD8^+^ Tex. These findings provide a unique and detailed perspective, which is helpful in changing this completely unpopular state of hypofunction and intensifying the effect of immunotherapy.

## 1 Introduction

T cells within the immune system patrol to find pathogens in the human body. When T cells turn into the tumor microenvironment (TME), they will detect and distinguish normal cells and cancer cells according to the tumor antigen information presented by major histocompatibility complex (MHC) molecules on antigen-presenting cells (APC) cells, which induces inflammatory response and antitumor response by secreting cytotoxic secretions (1). However, when antigens chronically and continuously stimulate CD8^+^ T cells over an extended period, CD8^+^ T cells will have exhausted key characteristics appearance, including decreased effector function and proliferation, elevated co-inhibitory receptor expression, reduced cytotoxicity, and changes in metabolism and transcription (2). Exhausted-like CD8^+^ T cells (CD8^+^ Tex) initially found in mice with chronic infection with the lymphocytic choriomeningitis virus (LCMV) (3). Since then, multiple research efforts have demonstrated that Tex is essential for chronic infection, tumors, and autoimmune diseases (2, 4). CD8^+^ T cells developing into CD8^+^ Tex will go through the following four stages: dormant Tex progenitor (Tex^prog1^), proliferating and circulating Tex progenitor (Tex^prog2^), circulating and slightly toxic intermediate exhausted T cells (Tex^int^), and terminally differentiated exhausted T cells (Tex^term^) (5). T cell exhaustion is triggered by the following four factors in the TME: 1) long-term antigen stimulation and high expression of co-inhibitory receptors/immune checkpoints; 2) soluble cytokines (type I interferon, IL-2, IL-10, and transforming growth factor-β (TGF-β)) in the TME; 3) recruitment of immunosuppressive cells: regulatory T cells (Tregs), Myeloid-Derived Suppressor Cell (MDSC), and tumor-associated macrophages (TAMs); 4) and the lack of oxygen and nutrients in the TME (6).

The successful outcome of cancer immunotherapy, particularly immune checkpoint blockade (ICB) and adoptive T cell treatment (ACT), is diminished by T cell exhaustion (7). Tex is the main reactive cell of ICB, and it is the breakthrough point in improving the efficacy of ICB. One of the aims of ICB therapy is expanding and infiltrating precursor exhausted T cell (Tpex) (8). In addition, TME induces dysfunctional Chimeric Antigen Receptor T (CAR-T) cells and T cell receptor-engineered T (TCR-T) cells, which are major obstacles to ACT in solid tumors. Reducing T cell exhaustion and intensifying the effectuality of ACT not only need to optimize chimeric antigen receptors and downstream signal transduction but also should intervene in transcriptional and metabolic disorders (9). Notably, CAR-Treg therapy, which induces Treg depletion, can also inhibit tumor development (10, 11).

Therefore, this review mainly focuses on the intercellular signaling networks interacting with Tex, the immunosuppressive cells in the TME, and the dendritic cells (DCs) assisting CD8^+^ T cells in anti-tumor immunity. In the end, we discussed how to deal with the challenges of Tex in ICB therapy and ACT.

## 2 Immune cells regulate T-cell exhaustion through intercellular communication

**Figure 1:**
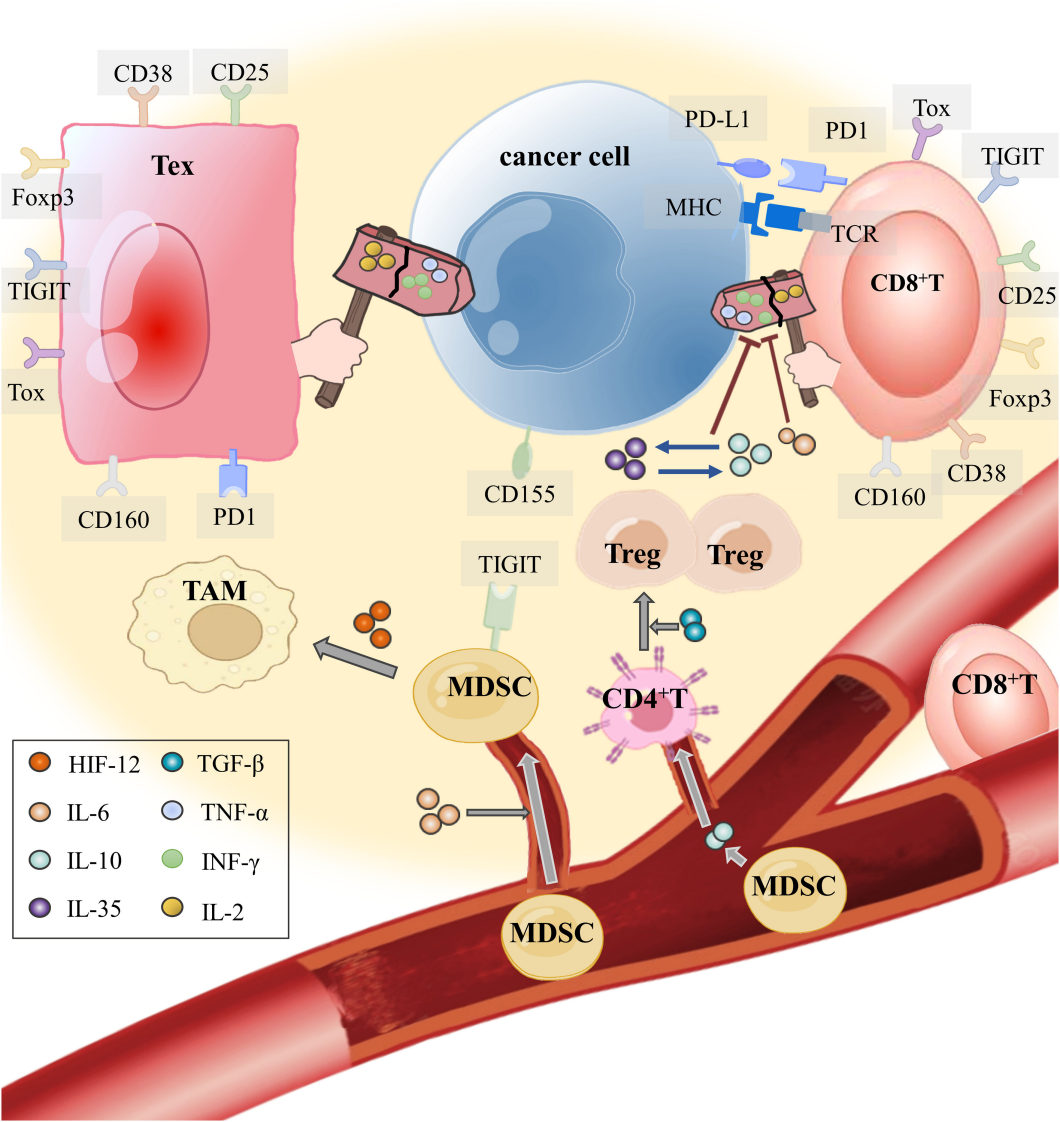
How do tumor cells instigate immune cells into the TME and make them accomplices? T cells in the TME, through their T cell receptor (TCR), recognize the antigen presented by MHC on the APC (including tumor cells) and secrete effector cytokines to exert antitumor immunity. IL-6, which is released by tumor cells in the TME, not only restrains the antitumor immunity of effector T cells but also recruits MDSCs into the TME. Hypoxia in the TME makes HIF-1 α highly expressed. With the mediation of hypoxia-inducible factor-1α (HIF-1α) and IL-6, MDSCs induce monocyte myeloid-derived suppressor cells (m-MDSCs) to differentiate into TAMs for obstructing T cell activation and effector response. In addition, IL-10 produced by MDSCs attracts CD4^+^ T cells to enter the TME and develop into Tregs with TGF-β. The interaction between IL-35 released by CD177^+^ Tregs and IL-10 released by PD-1^+^ Tregs inhibits CD8^+^ T cells from eliminating tumor cells and mediates T-cell exhaustion.

When neoplasm antigens are continuously exposed over an extended period, the development of CD8^+^ Tex integrates other immune cells expressing co-inhibitory or co-stimulatory receptors and cytokines transmitting inhibitory signals in the TME (6). We summarized the intercellular communication between Tregs, MDSCs, TAMs, DCs, and Tex (**Figure 1**).

### 2.1 Tregs

Tregs can maintain self-tolerance and avoid excessive immune response damage, but Tregs participate in tumor immune evasion mechanisms in the TME. Tregs in the TME can inhibit effector T cells (Teff) and myeloid cells from two aspects. On the one hand, it upregulates the immune checkpoint ligand of tumor cells to avert CD8^+^ T cell immunological inspection; On the other hand, it secretes cytokines to induce CD8^+^ Tex. Immunosuppressive cytokines (IL-10, IL-35, and TGF-β) produced by Tregs have the capacity to both drive CD8^+^ T cells to cease becoming Teffs and deliver immunological escape indications to tumor cells (12).

#### 2.1.1 IL-10 and IL-35

In tumor formation and metastasis, Tregs are the main source of IL-10, a kind of cytokine with durable and versatile anti-inflammatory effects (13). In the TME, Tregs significantly secrete more IL-10, which immune checkpoint molecule TIM-3 plays a non-negligible role. Banerjee H et al. found that Tregs upregulated the secretion of IL-10 promoting CD8^+^ Tex through T cell immunoglobulin and mucin domain-containing protein 3 (TIM-3) (14). Interestingly, IL-10 also reverses the process of inducing Treg differentiation in the TME, so that the interaction between IL-10 and Treg further acts on T cell exhaustion (15). Furthermore, it was discovered that Salkeni et al. thought IL-10 induced T cell stimulation in the TME. Thus, more investigation is required to learn how IL-10 influences T-cell exhaustion (16). Different Treg subsets produce different cytokines. In the study by Ma et al. of esophageal squamous cell carcinoma, PD-1^+^ Tregs were the primary producers of IL-10, whereas CD177^+^ Tregs were the primary producers of IL-35 in the TME (17). As a heterodimer of IL-12, IL-35 derived by Tregs regulates the generation of immune checkpoint molecules: programmed cell death protein 1 (PD-1), TIM-3, and lymphocyte-activation gene 3 (LAG-3), which promote T cell exhaustion by cooperating with IL-10 (12).

#### 2.1.2 TGF-β

An important modulator of both innate and adaptive immunity is TGF-β (18), which is released by tumor cells and immune cells (19, 20). TGF-β prevents immature T cells from differentiating into Th1 cells, promoting Foxp3 to encourage the differentiation of naïve CD4^+^ T cells into Treg cells and regulating TGF-β signaling to mediate the anti-tumor immune function of Treg inhibiting CD8^+^ T cells in the TME (21, 22). Knocking down endogenous TGF-β receptor type-2 (TGFBR2), blocking TGF-β signaling, or inhibiting the deubiquitination of Foxp3 from upregulating in the TME can inhibit the differentiation of Tregs in the TME and prevent Tex (23–26). In addition, colonic carcinoma metastasis signal mediated by TGF-β can stimulate the upregulation of Foxp3, CD25, and PD-1, which are markers of CD8^+^ Tex (27).

### 2.2 MDSC

In addition to facilitating tumor vascularization, MDSCs in the TME also aid in the tumor cells’ invasion and metastasis. MDSCs inhibit T cell activation and promote the differentiation process of Tregs. In addition, MDSCs can also polarize TAMs (28). In the TME, MDSCs recruitment and infiltration are accompanied by a decrease of Teff-produced cytokines, upregulation of multiple inhibitory receptors (IR), and alterations to T cell subsets, which undermine the long-term maintenance of tumor immunity (29).

#### 2.2.1 IL-6

IL-6, which can be produced by a variety of cells, is a classical cytokine regulating various inflammations. In the inflammatory response, it possesses both opposed- and anti-inflammatory characteristics (30). The aberrant production of IL-6 is the result of TME chronic inflammation (31, 32), so tumor cells will produce and secrete more endogenous IL-6 when proliferating, infiltrating, or metastasizing (33, 34). Tumor patients who showed higher concentrations of IL-6 in their blood in clinical had suppression of CD8^+^ T cells proliferation and cytokine generation, and they had higher adverse clinical outcomes (35). In the TME, IL-6 can bind its receptor, transmit signals through the downstream JAK/STAT3 pathway, and activate the transcription of tumor-related genes. In addition, IL-6/JAK/STAT3 signal transduction upregulates immune checkpoint ligand PD-L1 expressed and regulates MDSCs to suppress tumor immunity (36). MAPK and IL-6-mediated signaling pathways impact immature MDSCs (i-MDSCs) in the TME, which are activated by IL-6 and attracted to tumor locations where they develop into mature MDSCs and decrease tumor immunity (37). IL-6 recruits MDSCs in the TME, which can inhibit T cell activity and promote partial CD8^+^ Tex through IFN-γ (38). One important regulator in preserving MDSC differentiation, proliferation, and function is IL-6. When MDSC inhibitors are applied to inhibit the number and function of MDSC, the expression of IL-6 in the TME is also inhibited, and the active immune response is re-established (39). It’s interesting to note that IL-6 released by MDSCs was linked to multidrug resistance via the IL-6/STAT1 axis in the work by Dhar S et al. (40). Within the congenital drug resistance model, combining treatment of Cytotoxic T-Lymphocyte Associated Antigen 4 (CTLA-4) inhibitor and BET inhibitor induced the decrease of MDSCs infiltration and downregulated TIM-3 and LAG-3 in the TME (41). IL-6 in the TME recruits MDSCs to participate in the drug resistance of tumor therapy and may be related to CD8^+^ Tex, but the clear mechanism needs further exploration.

#### 2.2.2 Immune checkpoint

The reason why acquired resistance happens to ICB therapy is that MDSCs induce CD8^+^ Tex. According to Koh J et al., the greater the infiltration of MDSCs with the progression of the patients’ non-small cell lung cancer, MDSCs inhibited T cell activity and induced T cell exhaustion in the TME, which contributed to resistance in anti-PD-1 therapy (42). Additionally, Galectin 9 (Gal-9) acts as an immune checkpoint ligand for TIM-3. MDSCs upregulate the expression of Gal-9 in the TME. A rise in Gal-9 causes CD8^+^ T cells to overexpress TIM-3, which prevents T cells from secreting effector cytokines. Thus, the TIM-3/Gal-9 route may be used by MDSCs to promote CD8^+^ T cell exhaustion (43). In addition, clinical research on the treatment of breast cancer showed that CDK4/6 inhibitors avoided the T cell exhaustion phenotype by reducing the expression levels of multiple immune checkpoints and the quantity of MDSCs (44).

#### 2.2.3 MDSCs interact with Tregs

As mentioned above, the primary source of IL-10 in the TME is Tregs. It is undeniable that cytokines including IL-10 released by MDSCs can activate Tregs and TAMs in the TME (45). MDSCs and Tregs in the TME share common microRNAs related to immune regulatory pathways, including TNF, TGF-β, FOXO, and Hippo (46, 47). Therefore, MDSCs and Tregs not only have the effect of inhibiting tumor immunity, but also interact and cooperate with each other in TME.

Following the injection of carboplatin, MDSCs stimulated IL-13/IL-33 via the VCAM/RANTES pathway, facilitating CD4^+^ T cells to convert into Tregs and encouraging Tregs aggregation at tumor locations. The inhibitory receptor TIGIT was upregulated by CD155. The expression of TIGIT was favorably linked with the ratio of T cells to MDSCs in TME. CD8^+^ T cells upregulated the expression of CTLA-4, TIM-3, and CD160 (48). Blocking TIGIT/CD155 signal transduction alleviates Tex and delays tumor growth (49). MDSCs recruited in the TME, mediate the growth and metastasis of tumors through phosphatidylinositol-3-hydroxylase (PI3Kγ), inhibiting the transcription of down-regulated T cell exhaustion genes in mouse tumors with PI3Kγ, and reducing infiltrated Foxp3^+^ Tregs in the TME (50).

#### 2.2.4 MDSC with TREM1

The triggering receptor expressed on myeloid cell 1 (TREM1) is a key mediator of innate immunity (51). The high expression of TREM1 in polymeric myeloid-derived suppressor cells (PMN-MDSCs) and M2-like macrophages is associated with the inferior prognosis of lung cancer (52), liver cancer (53), renal cell carcinoma (54), breast cancer (51)and other tumors. Knockdown of TREM1 can enhance the therapeutic effect of anti-PD-1, probably by inhibiting the function of MDSCs and T cell exhaustion in the TME (55). In the DLL3-TREM1/DAP12 CAR-T (DLL3-DT CAR-T) therapy developed by Nie F et al., DLL3-DT CAR-T cells have durable antitumor responses and increased production of memory T cells in the TME (56).

#### 2.2.5 MDSC with exosome

Increasing evidence suggests that exosomes can promote chemoresistance within the TME (57). Tumors regulate the TME by secreting microRNAs from exosomes. Pancreatic cancer-derived exosomes have a macrophage migration inhibitor factor (MIF), and MIF tautomerase regulates the expression of genes required for the differentiation, recruitment, and activation of MDSCs (58). In addition, exosome mir-1298-5p promotes the immunosuppressive effect of MDSCs, promoting the growth of neuroglioma (59). Interestingly, MDSC−derived exosomes (MDSC exo) are hyperactivated inducing CD8^+^ T cell exhaustion and promoting the production of reactive oxygen species to meet the oxygen supply of tumor cells (60).

### 2.3 TAMs

Tumor cells in the TME recruit TAMs and induce M2 polarization by secreting cytokines and creating an immunosuppressive environmental state (61). Liu C et al. found that APOE^+^ macrophages are closely connected to CD8^+^ Tex (62), which means TAMs inhibit antitumor T‐cell immunity in solid tumors. In the study of Yin C et al., hepatocellular carcinoma (HCC) induced M2 polarization by upregulating the expression of miR-146a-5p in exosomes and activating NF-κB signaling to induce proinflammatory factors for remodeling macrophages. Blocking the interaction between the transcription factor Sal-like protein-4 (SALL4) and mir-146a-5p reduces the expression of inhibitory receptors and reverses the exhaustion of CD8^+^ T cells (63). In addition, M2-like macrophages secrete extracellular vesicles to promote CD8^+^ T cells exhaustion of HCC through the mir-21-5p/YOD1/YAP/β-catenin pathway (64).

#### 2.3.1 Hypoxia

TAMs are abundant in TME, tumor blood vessels, and stromal regions, which have immunosuppressive effects and promote Tex (61). Hypoxia is frequently seen in TME, and there is a high expression of hypoxia-inducible factor-1α (HIF-1α). HIF-1α can induce monocyte myeloid-derived suppressor cells (m-MDSCs) to differentiate into TAMs through IL-6/STAT3/p-STAT3 and inhibit T cell activation and effector function (65, 66). Targeted STAT3 tumor immunotherapy reverses the polarization and immunosuppression of TAMs in the TME (67, 68). Through the regulation of XOR-IDH3α, TAMs may impact the function of TAMs and CD8^+^ T cells. According to Lu Y et al. of hepatocellular carcinoma and xanthine oxidoreductase (XOR), within hypoxia TME, enzymes involved in macrophage polarization interact with IDH3α to mediate TAMs polarization and inhibit anti-tumor immunity of CD8^+^ T cells (69). The upregulation of CD8^+^ T cell immune checkpoints (PD-1, CD38, TOX) was positively correlated with the abundance of TAMs in the TME. In addition, Kersten K’s team also found that exhausted T cells would recruit monocytes to the TME and form TAMs to induce CD8^+^ T cell exhaustion, further inhibiting anti-tumor immunity (70).

HIF-1α can not only induce m-MDSCs to differentiate into TAMs but also upregulate the expression of PD-L1 on the surface of MDSCs by binding to PD-L1 receptor on the surface of T cells, which induces Tex (71, 72). In a therapeutic study of nanomaterials transporting catalase into the TME, ROS were increased by degrading H_2_O_2_, significantly reducing MDSCs and Tregs (73).

#### 2.3.2 Chemokines

Chemokines which are a kind of guiding cell migration cytokines expressed in the TME will recruit immune cells into the TME (74–77). There are many different chemokines produced by different cells in the complex TME. In the study of Kamat K et al., CCL23 secreted by TAMs induced T cell exhaustion by mediating GSK3β to upregulate T cell exhaustion markers in ovarian cancer (78). In this part, we focus on the chemokines related to T cells exhaustion and explore their potential research directions in ICB and ACT therapy.

As mentioned above, hypoxia in the TME induces the polarization of TAMs and the exhaustion of T cells (65). Several studies have shown that TAMs play an immunosuppressive role in the TME through the CCR5-dependent signaling axis (79–81), which can block the CCR5-related pathway to inhibit tumor invasion and metastasis (82). In advanced cancer patients, the proportion of exhausted T cells in a hypoxic environment is significantly increased. Analysis of exhausted T cells ligand-receptor interactions revealed that tumor cells would recruit T cells into hypoxic TME through the chemokine CCL3/CCL4/CCL5-CCR5 axis (83). Hernández-Verdin I et al. found that the increased expression of activation-induced cytidine deaminase (AID) caused by mutation induced T cells exhaustion by upregulating the expression of CXCL13/CCR5. The application of ICB therapy can block it, thereby reactivating exhausted T cells (84). Coincidentally, the study of Liu Z et al. found that RUNX3 was the key mediator of decitabine improving anti-PD-1 immunotherapy, and knockdown of RUNX3 significantly reduced the levels of CCR3 and CCR5 (85). In addition, TAMs in the TME will conduct cellular and molecular communication with immature DCs induced by tumor cells through CCR5/CCL5 molecules, which will destroy the antigen-presenting function of DCs (86). Similarly, the study of Horie M et al. also showed that HIF-1α would promote the recruitment of TAMs and DCs in the TME under hypoxia, and create an immunosuppressive microenvironment by upregulating the expression of CCR5/CCL5, inducing T cells exhaustion and peritoneal dissemination of cancer cells (87). TAMs in TME not only inhibit the anti-tumor effect of T cells, but also destroy the auxiliary function of DCs in this process. How DCs become a good helper of CD8^+^ T cells will be described in the next section.

Hedgehog (Hh) signaling in myeloid cells is essential for M2 polarization of TAMs and tumor growth. Tumor cells secrete an Hh ligand (SHH) to drive TAMs to polarize towards M2 (88). The production of CXCL9 and CXCL10 by polarized TAMs is inhibited, blocking the CXCL9/10/CXCR3 axis and inhibiting the recruitment of CD8^+^ T cells to the TME (88). Compared with normal renal tissue, the mRNA expression of CXCR3/CXCL9/10/11 in renal cell carcinoma was significantly increased (89), and the upregulation of CXCR3 expression in renal cell carcinoma was related to the hypoxic state in TME (90). In addition, the study of Azuma M et al. demonstrated that Poly (I: C) interacted with DCs, cross-upregulated the expression of CXCR3, and promoted the recruitment of CD8^+^ T cells to the TME (91). However, CD8^+^ T cells recruited to the TME will gradually downregulate the expression of CXCR3 under chronic antigen stimulation, while upregulating the exhaustion markers PD-1 and LAG-3. In addition, the knockdown of CXCR3 can enhance the production of IFN-γ, an effector molecule of CD8^+^ T cells, indicating that CXCR3 promotes the loss of effector function and the process of T cell exhaustion (92). Interestingly, the expression of CXCR3 in tumor mice that did not respond to ICB therapy was significantly reduced (93). Enhancing the CXCR3/CXCL9 axis can promote the interaction between DCs and CD8^+^ T cells in the TME and improve the sensitivity to PD-1 blockade and clinical therapeutic efficacy (94). The enhancing effect of CXCR3 in PD-1/PD-L1 therapy may be related to its ability to recruit T cells into the TME in an antigen-independent manner and to activate the bystander memory T cells (95). At the same time, previous studies have shown that the CXCR3 ligand helps T cells and B cells recruited to the TME form a cell network directly contacting each other. This network contains TCF1^+^ PD-1^+^ CD8^+^ T cell progenitors, which can be transformed into cytotoxic CD8^+^ T cells during ICB treatment (96, 97). Therefore, Sullivan PM et al. demonstrated that overexpressed CXCR3 can be applied to ACT therapy to drive the trafficking of CD8^+^ T cells into the TME and synergize with PD-1 checkpoint blockade immunotherapy (98). In addition, although it played an important role in the entry of CD8^+^ T cells into the TME, they gave an explanation for the incomprehensible result that CXCR3 expression was downregulated in the TME: it may be attributed to cell-extrinsic variables in the TME, such as inhibitory receptor signal transduction and TGF-β secreted by tumor cells (99). Similarly, Li A et al. demonstrated that a unique anti-human GARP antibody (named PIIO-1) treatment could block CD8^+^ T cell exhaustion by reducing TGF-β signaling in the TME while enhancing CD8^+^ T migration into the TME in a CXCR3-dependent manner (25).

#### 2.3.3 Interferon regulatory factor 8 (IRF8)

As mentioned above, tumor cells secrete Shh to drive TAMs to polarize towards M2, and the production of CXCL9 and CXCL10 by polarized TAMs is inhibited (88). In the study of He N et al., it was found that the polarization of TAMs in microwave ablation (MWA) therapy was mediated through the nuclear factor-κB/JAK-STAT1 signaling pathway. MWA significantly upregulated the expression of IFN-γ stimulated transcription factors (especially IRF8) (100). Among the nine IRF members regulating interferon signaling, the expression of IRF8 in HCC was associated with inferior prognosis of HCC patients (101). In addition, MWA combined with αPD-L1 treatment promoted the production of CXCL9 and blocked IFN-γ/CXCL9/CD8^+^ T axis which could promote tumor progression (100). Tumor cells will induce the expansion of TAMs, which requires TAMs to express IRF8. Specific deletion of IRF8 in TAMs blocks T-cell exhaustion and inhibits tumor growth (102). Therefore, TAMs participate in the process of T cell exhaustion by upregulating IRF8. The study of Wu H et al. demonstrated that IRF8 downregulated the expression of CCL20 by inhibiting c-fos transcription and mediated the immunosuppressive effect of TAMs recruited in the TME (101).

### 2.4 DCs

In the TME, immunosuppressive cells Tregs, MDSCs, and TAMs cooperate with each other, but CD8^+^ T cells are not in a helpless situation. DCs will cooperate to participate in the anti-tumor immunity in the TME and help the anti-tumor immunity of CD8^+^ T cells through antigen presentation (103). The crosstalk between DCs and T cell exhaustion is mainly concentrated on interferons (type I interferons and type II interferons) and IL-2. The dual role of IL-2 still needs further exploration.

#### 2.4.1 Interferon

##### 2.4.1.1 Type I interferon

As antigen-presenting cells, DCs must go through the following processes to achieve effective anti-tumor immunity: professional antigen-presenting cells (pAPCs) absorb tumor neoantigens and promote the maturation and development of immature T cells through cross-presentation. This process is mainly participated by DCs (103). According to the dynamic changes of TME, DC subsets (conventional type 1 dendritic cells (cDC1), conventional type 2 dendritic cells (cDC2), plasmacytoid dendritic cells (pDCs)) affect the progression of tumors through various mechanisms (104). Among them, cDC1 is essential for anti-tumor immunity, which allows tumor antigens to be presented cross-presentatively to activate T cells (105). cDC2 has also been shown to drive protective anti-tumor CD4 T cell immunity (106). pDCs can produce type I interferon in the TME to promote the maturation of cDC1 and type I interferon also enhances the ability of CD8^+^ T cells (107, 108). However, TME also induces pDCs to express immunosuppressive molecules to encourage the formation of tumors. Chemotherapy carboplatin-resistant tumor cells recruited pDCs and upregulated pDC immune checkpoint ligand inducible costimulator ligand (ICOS-L) expression to maintain MDSCs-dependent immunosuppressive ability and promote Tex in the TME (48). Excessive type I interferons produced by pDCs also drive T-cell exhaustion. According to Wu T et al., the transcription factor TCF-1 induced B-cell lymphoma 6 (BCL6) to antagonize IFN-α/β signaling mediated Tex and maintain the stem cell properties of T cells (109).

##### 2.4.1.2 Type II interferon

After sensing the production of IFN-γ by neighboring T cells, activated DCs produce IL-12 to stimulate anti-tumor immunity (110). The specific recognition of antigen peptides on tumor cells by CD8^+^ T cells require the presentation of major histocompatibility complex (MHC or HLA) class I molecules. IFN-γ signaling is a key pathway to regulate the expression of HLA-I and HLA-II, and its destruction is one way that tumor immune evasion works (103). HLA-DR is a class II MHC molecule expressed by CD8^+^ T cells. After being stimulated by tumor antigens, T cells show a multitude of cytokines including IFN-γ, TNF-α, and IL-2 (111). The defect in IFN-γ production means that T cells have entered the exhaustion phase (112).

#### 2.4.2 IL-2

IL-2 is a significant cytokine that regulates Tex. IL-2 in the TME is mainly secreted by CD8^+^ T cells and activated DCs. IL-2 exerts its immunomodulatory effect mainly by binding to IL-2R. Various conformations of IL-2R mediate the downstream effects of IL-2. Due to the variable conformation of IL-2R, IL-2 shows a dual role of both immunostimulatory and immunosuppressive, which is related to interleukin-18 (IL-18) and Tregs.

##### 2.4.2.1 Immunostimulatory effect of IL-2

Reduced IL-2 production is considered an early sign of CD8^+^ Tex. DCs and CD8^+^ T cells secrete IL-2, which can also affect the anti-tumor effect of CD8^+^ T cells. According to Zhang et al., histamine H1 receptor (H1R1) antagonists can stimulate T cell activation and promote IL-2 secretion, thereby inhibiting T cell exhaustion clinically related to high expression of H1R1 (113). In addition, IL-2 antagonizes TOX by driving STAT5 activity to exert anti-tumor immunity that reverses CD8^+^ Tex (114). In a study of CAR-T cell therapy combined with infusing aAPC, aAPC infusion promoted the specific recognition of CAR-T cells to tumor cells and released more IFN-γ, TNF-α, and IL-2. The cytokine mixture IL-2-9-21 not only inhibits the upregulation of the immune checkpoints on CAR-T cells but also inhibits the PI3K/AKT signaling pathway and enhances the JAK/STAT3 signaling pathway. This prevents the T cell exhaustion made by the CAR-T cells’ constant binding to tumor antigens in the TME (115).

##### 2.4.2.2 Immunosuppressive effect of IL-2

A high concentration of IL-2 in the TME will activate the initial multiplication of naive T cells. The activated CD8^+^ T cells transiently express IL-2Rαβγ, a high-affinity receptor for IL-2, and then are driven by IL-2 to differentiate into CD8^+^ T cells that continuously express the medium affinity receptor IL-2Rβγ (116). Due to the different conformations of IL-2R, IL-2 also exerts immunosuppressive effects. In the study of Feriz AM et al., tumor cells stimulate immature DCs to migrate to the tumor site through inflammatory chemokines. DCs infiltrating the TME upregulate many signaling pathways, such as TNF-α/NF-κB, IL-2/STAT5, and E2F, while the IL-2 autocrine signal in DCs activates the JAK/STAT5 pathway to induce apoptosis of mature DCs (86). IL-2 can also induce the STAT5/TPH1/5-HTP/AhR pathway in CD8^+^ T cells, leading to synergistic upregulation of inhibitory receptors and downregulation of effector molecules, which induces CD8^+^ Tex appearance in the TME (117).

##### 2.4.2.3 IL-2 and Interleukin-18 have dual immunomodulatory

In addition, CD8^+^ Tex can be induced by IL-18 in the TME via the IL-2/STAT5/mTOR pathway. IL-18 may promote the deterioration of pancreatic cancer (118). Conversely, in a study of CAR-T cell therapy targeting Delta-like protein 3 (DLL-3), IL-18 released by CAR-T cells inhibited the upregulation of immune checkpoints PD-1, TIGIT, LAG3, and TIM-3 of DCs (119). IL-18 production increases CAR-T and CD8^+^ T cells’ activation. We believe that IL-2 may have a role in the dual function of IL-18 in tumor immunity, and further in-depth study is needed.

##### 2.4.2.4 IL-2 and Tregs have dual immunomodulatory

IL-2 high-affinity receptor (IL-2Rαβγ) is continuously expressed on Tregs. Low levels of IL-2 selectively bind to the high-affinity receptor IL-2Rαβγ of Tregs but do not activate T cells in the TME. Foxp3 and CD25 expression of Tregs need to be maintained by IL-2. Because it cannot produce IL-2, Tregs compete with CD8^+^ T cells to deplete IL-2 in the TME, thus preventing the anti-tumor immunity of CD8^+^ T cells (120). As mentioned above, the impaired effector function of CD8^+^ T cells in the TME and the appearance of exhausted phenotype are positively correlated with the accumulation of Tregs. The study of Noyes D et al. demonstrated that Tregs mediated the depletion of IL-2 in the TME and further exacerbated the exhaustion of CD8^+^ T cells (121). However, in a clinical study of Dasatinib on chronic myelocytic leukemia (CML), patients taking Dasatinib had significantly reduced plasma IL-2 levels, suppressed STAT5 phosphorylation in Tregs cells, and TIM-3-mediated exhaustion of CD8^+^ T cells (122). How to stimulate CD8^+^ T cells without inducing TME Tregs has proven a significant obstacle in the development of anticancer treatments targeting IL-2.

## 3 Immune cell-CD8^+^ Tex crosstalk in cancer immunotherapy

**Figure 2:**
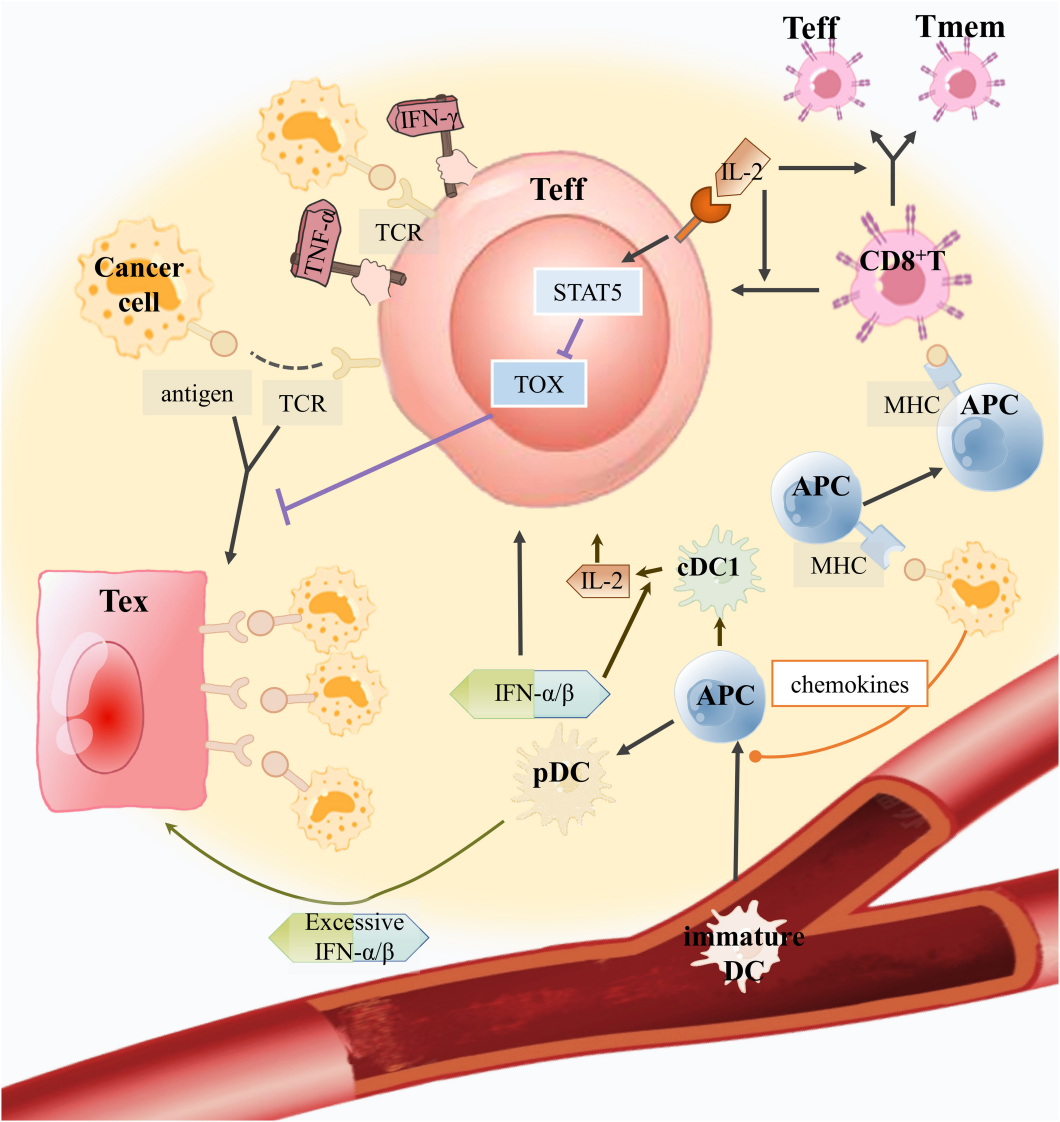
How can good helpers of CD8^+^ T cells inhibit Tex and assist anti-tumor immunity in the TME? Immature DCs can sense chemokines secreted by tumor cells to develop into APCs and enter the TME to recognize tumor antigens. Tumor antigens are expressed by APC, which further stimulates CD8^+^ T cells to develop into Teff and memory T cells (Tmem). Plasmacytoid pre-dendritic cells (pDCs) secrete IFN-α/β, which can improve Teff activity and encourage the maturation of APCs into conventional dendritic cell 1(cDC1) to produce IL-2. IL-2 produced by cDC1 will bind to the IL-2R on Teffs to mediate the STAT5/TOX pathway to inhibit excessive antigen-stimulated Teffs toward exhaustion. In addition, IL-2 can also encourage CD8^+^ T cell growth. However, excessive IFN-α/β production by pDCs would promote Teff toward CD8^+^ Tex.

Recently, although emerging immunotherapy has made indisputable great progress in clinical application, there are also some cases of poor therapeutic effect. T-cell exhaustion directly affects the efficacy of ICB therapy and ACT therapy. We summarize the role of immune cells in the TME-CD8^+^ Tex in ICB and act to better cope with the challenges of complex TME (**Figure 2**).

### 3.1 ICB

ICB therapy has made great progress in tumor treatment, but it is only effective for a small number of patients. There are many factors that affect ICB therapy, among which the interference effect of the immunosuppressive state composed of immunosuppressive cells (Tregs, MDSCs, and TAMs) in the tumor microenvironment cannot be ignored. Exhausted CD8^+^ T cells showed up-expression of co-inhibitory receptors (CTLA-4, PD-1, TIM-3, LAG-3, etc.) and downregulation of co-stimulatory receptors (CD28, 4-1BB) (123–125). The co-stimulatory signal is lacking, and the co-inhibitory signal is too strong. Immune cells, which are also engaged in the process of ICB inhibiting Tex, also exhibit significant expression of these inhibitory receptors. In addition, we also summarized the common CAR-T targets in tumors and the combination therapy with ICB (**Table 1**).

**Table 1:** The common CAR-T targets in tumors and the combination therapy with ICB.

Solid Tumor Classify		Normal CAR-T Target	Combine ICB Target research	Reference
Thoracic tumors	Lung Carcinoma	EGFR	PD-1, PD-L1	(126–130)
		ROR1	PD-1, PD-L1	(131)
		EphA2, B7-H3	N/A	(132–135)
	Breast Carcinoma	HER2	PD-1, PD-L1	(136–138)
			CTLA-4	(139)
		ROR1, KRAS, CEA, EphA2, Nectin-4, MUC1, c-Met, EpCAM	N/A	(140–142)
Gastrointestinal tumors	Gastric Carcinoma	HER2, Claudin 18.2	N/A	(143, 144)
	Hepatocellular Carcinoma	GPC3, AFP, EpCAM	N/A	(145–147)
	Colorectal Carcinoma	CEA	TIGIT	(148)
		GUCY2C	N/A	(149)
	Pancreatic Carcinoma	MSLN, Mesothelin, Claudin 18.2	N/A	(150–153)
Tumors of the reproductive system	Ovarian Carcinoma	MSLN, MUC1	N/A	(154, 155)
Tumors of the urology system	Renal Cell Carcinoma	CA9	N/A	(156)
	Prostatic Carcinoma	PSMA	N/A	(157)
Other tumors	Neuroblastoma	CD171	N/A	(158)
	Glioblastoma	EGFRvIII, CD133	PD-1, PD-L1	(159, 160)

N/A, Not Applicable.

#### 3.1.1 CTLA-4

CTLA-4 is mainly expressed in activated T cells, but also in Tregs (161). CTLA-4 binds to CD80/CD86 on the tumor surface to prevent T cell activation in the TME (121). As mentioned earlier, the exhaustion-like phenotype and the impairment of effectors of CD8^+^ T cells are positively correlated with the accumulation of Tregs. Tregs have a high level of CTLA-4 expression, which is essential for developing and maintaining the exhaustive phenotype. In addition, inhibiting CTLA-4 signaling can inhibit the recruitment of Tregs and relieve the immunosuppressive effect of Tregs. Ipilimumab and tremelimumab are FDA-approved CTLA-4 inhibitors for clinical use (162, 163). CTLA-4 inhibitors have the ability to inhibit CTLA-4’s binding to CD80/CD86, promote T-cell proliferation, and have anticancer effects (164).

#### 3.1.2 PD-1

The surface of activated T cells, NK cells, DCs, and other cells expresses PD-1. PD-L1 is highly expressed in tumor cells, and the combination of the two mediates tumor immune escape. PD-1 in TME can not only mediate TCR to decide the activation and differentiation of T cells but also activate Tregs. Therefore, PD-1 plays a non-negligible role in the anti-tumor immunity of remodeling the TME (165, 166). PD-1 is upregulated on T cells of renal angiomyolipoma and pulmonary lymphangioleiomyomatosis. Blocking both PD-1 and CTLA-4 together is more efficient than targeting just one PD-1 inhibitor (167). After the combined blockade of PD-1 and CTLA-4 in the orthotopic hepatocellular carcinoma model, PD-1 was expressed in moderate amounts on Tex^prog^, and Tex^term^ almost disappeared in the TME. Hence, the combined treatment downregulated PD-1 expression and reversed the anti-tumor immunity of Tex (168). These studies confirm that combination therapy is better at reducing CD8^+^ Tex. Although the efficacy of combination therapy is better, the immune-related adverse reactions (irAEs) related to ICB drugs are more serious (169). Therefore, a significant issue with ICB therapy is how to balance its effects with the emergence of irAEs, and more research is needed. In addition, whether PD-1 inhibitors combined with other immunotherapies can reverse T-cell exhaustion is a hot topic of future research (170–172).

#### 3.1.3 TIM3

TIM3 is expressed in Tregs, TAMs, DCS, natural killer cells (NKs), and mast cells. TIM3 is a key cytokine for Tex^term^ and is at the junction of T-cell exhaustion and rejuvenation (124, 173). The interaction between TIM3 and galectin 9 (Gal9) in CD8^+^ T cells leads to the reduction of the production of cytokines (IFNγ, IL-2, and TNFα), the inhibition of T cell proliferation, and the inhibition of anti-tumor immunity (174). The progression of tumors can be effectively controlled by simultaneously inhibiting TIM3 and PD1. Datar I et al. indicated that lung cancer patients with TIM3^+^ CD68^+^ TAM had shorter survival and worse prognosis than those with TIM3^-^ CD68^+^ TAM (175, 176). TIM3^+^ Treg is also more immunosuppressive than TIM3^-^Treg (177). In addition, the lack of TIM3 on DCs promotes the proliferation of CD8^+^ T cells in the TME (178).

#### 3.1.4 LAG-3

LAG-3 is an inhibitory receptor protein on activated T cells, NK cells, B cells, plasma cells, and DCs. In the study of Kano Y et al., the soluble recombinant protein LAG-3-Ig, which inhibits the LAG-3 signal, was combined with a tumor vaccine, and it was found that they could down-regulate the expression of LAG-3 and other co-inhibitory receptors on CD8^+^ T cells, prevent Tex, and boost the tumor vaccine’s therapeutic impact in concert (179). The expression level of LAG-3 increases with the stimulation of tumor antigens. Under long-term stimulation, LAG-3 and other co-inhibitory receptors are continuously expressed on T cells and mediate the exhaustion of T cells (180). Research has demonstrated that LAG3 inhibition can change Tex’s activity. In comparison to single inhibition, dual inhibition of PD-1 and LAG3 produces higher T-cell activation and reduces Tex (181, 182).

### 3.2 ACT

ACT refers to the transformation, expansion, and quality inspection of immune cells isolated from patients *in vitro*, and then reinfused into patients to play an anti-tumor role. The ACT includes CAR-T and TCR-T, and we emphasize CAR-T therapy. Tex is a main challenge affecting the therapeutic effect of CAR-T (183). CAR-T cell therapy can be improved by enhancing chimeric antigen receptors and concentrating on how immune cells affect CAR-T cells (184).

#### 3.2.1 CAR-T

Recent years have seen a remarkable development in CAR-T cell therapy. By collecting isolated T cells, and integrating antibodies targeting cells, CAR-T cells are generated and reinfused into patients to kill tumor cells (184). CAR-T can induce the remission of hematological system tumors effectively, but there is still a problem of recurrence in some patients (185). When it comes to treating solid tumors, the therapeutic effect of CAR-T cells is limited, which is characterized by poor penetration, persistence, and low proliferation ability. Therefore, choosing a target has been a hot subject in CAR-T cell therapy research (186–188). CAR-T cells and endogenous CD8^+^ T cells have similar gene expression and chromatin accessibility, so CAR-T cells will also be stimulated by sustained antigens during anti-tumor immunity, driving CAR-T cell exhaustion (189). Furthermore, the immunosuppressive cells also take part in the CAR-T cells’ exhaustion (23, 190).

##### 3.2.1.1 CD4^+^/Treg-CAR-T

Tregs may encourage CD8^+^ T cells’ exhaustion. The immunosuppressive effect mediated by Tregs is often applied to autoimmune diseases (191). Recently, CAR-Treg with antigen specificity has emerged, which is similar to the role of Treg (11). Interestingly, CAR-Tregs that are chronically stimulated by antigens will also have a similar exhaustion state and loss of effector function (10). Tregs as well as CD8^+^ T cells showed exhaustion-related elevation of immunological checkpoint molecules (165, 192). Luo Y et al. indicated that the neoadjuvant treatment of ovarian cancer with PARP inhibitor (PARPi) can exhaust Tregs in the TME and inhibit tumor growth (193). Treg exhaustion may lead to the development of a novel anti-tumor immune cell therapy, despite the fact that Tregs have the impact of suppressing anti-tumor immunity in the TME. Increased effector T cells and induction of Tregs can be seen in gut‐associated lymphoid tissue, which leads to improved clinical outcomes of cancer immunotherapy with lower incidence of immune‐related adverse events (194).

It is well known that Tregs is one of the cell subsets produced by CD4^+^ T cell differentiation. Boulch M et al. used the functional intravital image to find that anti-CD19^+^ CD4^+^ CAR-T cells mainly derived IFN-γ that diffuses in the TME and directly acts on tumor cells in treatment-responsive B-cell lymphoma (195). At the same time, Kruse B et al. found that TRP-1 CD4^+^ T cells isolated from ACT mice receiving CD4^+^ T could reprogram the myeloid cells network in the TME, which means IFN-γ produced by CD4^+^ T and myeloid cell-derived iNOS produced a tumor-killing phenotype through a synergistic effect (196). In addition, CXCL13^+^ Th cells, another cell subset differentiated by CD4^+^ T cells, and DC cells were shown to reactivate exhausted progenitor cells to effective antitumor CD8^+^ T cells after PD-1 blockade (197). In a recent study, CD4^+^ T cells combined with effector CD8^+^ T on the same DC cells to form a three-cell-type cluster to promote the toxicity of CD8^+^ T cells and eliminate tumor cells (198). The specific combination of this triad in TME will affect the clinical response of patients in ICB treatment (198). Therefore, in-depth understanding and research on the functions and regulatory mechanisms of CD4^+^ T cells will help to develop new CAR-CD4^+^ T anti-tumor therapies.

##### 3.2.1.2 MDSC and CAR-T

The recruitment of MDSCs will promote the development of Tex. Cytokines can modify CAR-T cells, which can inhibit MDSCs and improve therapeutic efficacy. CAR-T cells modified by different cytokines can inhibit MDSCs and enhance the therapeutic effect. In the study of Sun et al., Olaparib may inhibit the release of SDF1 α through HIF-1α, further restrict the recruitment of MDSCs to breast cancer tissues, reduce T-cell exhaustion, and raise CAR-T cell effectiveness (199). Next, Sun et al. also discovered that CAR-T cells modified with CXCR4 inhibited the recruitment of MDSCs through the STAT3/NF-kB/SDF-1α axis, enhancing the therapeutic effect in pancreatic cancer (200). Similarly, Liu et al. found that CAR-T targeting fibroblast activating protein (FAP) can also inhibit MDSCs recruitment, increase Tex, and improve pancreatic cancer (152). IL15Rα is preferentially expressed in MDSCs of glioblastoma, and IL15R-modified CAR-T cells can dual target MDSCs and tumor cells (201). Moreover, targeting TREM2 on MDSCs and TAMs can improve anti-PD-1 therapy’s effectiveness. Chen et al. created CAR-T cells secreting PD-1-TREM2 single chain variable fragment (scFv), which has a good effect on lowering the recruitment of MDSCs and TAMs in colorectal cancer (202).

##### 3.2.1.3 TAM and CAR-M

CAR-T cells can mediate the elimination of TAMs and increase CD8^+^ T cells’ expression. Recently, CAR-modified macrophage (CAR-M) therapy has garnered a lot of interest. Brown BD et al. constructed CAR-T cells targeting pan-macrophage marker F4/80, secreted IFN-γ, promoted the upregulation of MHC molecules on cancer cells and myeloid cells, effectively killed TAMs, and increased the activity and proliferation of CD8^+^ T cells (203). Rodriguez-Garcia A et al. indicated that CAR-T cells mediated the elimination of TAMs expressing folate receptor β, increased CD8^+^ T cells, and inhibited ovarian cancer growth (204). Human macrophages are altered by particular CARs to increase their phagocytic activity and ability to deliver antigens to malignancies. Compared with CAR-T cells, CAR-M can penetrate deeper into tumors, promote antigen presentation, and enhance the effect of CD8^+^ T cells (205).

##### 3.2.1.4 DC and CAR-T

CAR-T will increase DC infiltration and increase CD8^+^ T cells. Conversely, the DC vaccine will also improve CAR-T cell effectiveness (206). CAR-T promotes the phagocytosis of DCs and triggers the initiation of endogenous CD8^+^ T cells. During this process, CAR-T secretes IFN-γ, and its metabolism gradually tends to oxidative phosphorylation (207). Sun et al. combined the DC-tumor fusion vaccine with CAR-T cells. DC-tumor fusion vaccine enhanced the number of CD8^+^ T cells, attenuated T-cell exhaustion, and enhanced the therapeutic effect in solid tumors (208).

#### 3.2.2 TCR-T

TCR-T is to transfer the TCR gene sequence into T cells through genetic engineering technology, specifically recognizes tumor antigens, and inhibits tumors (184). Similar to the problems faced by CAR-T cells in the TME, TCR-T cells lack long-term persistence and are limited by immunosuppressive cells in the TME, resulting in limited therapeutic effect and TCR-T cell exhaustion (209). According to Cianciotti BC et al., knocking down the co-inhibitory receptors TIM-3 and LAG-3 connected to T cell exhaustion can resist the depletion of TCR-T cells and restore its antitumor effect (210).

## 4 Discussion and conclusion

T cell exhaustion may be an adaptive response to prevent cell death induced by overstimulation and activation in the TME and is regulated by multiple mechanisms during tumorigenesis. In the TME, immune cells (Tregs, MDSCs, and TAMs) regulate intercellular information transmission, inhibit the inflammatory microenvironment and regulate the expression of cell surface-related molecules, maintain the dynamic changes of the TME, and weaken the anti-tumor immune effect, metabolism, transcription and epigenetic regulation of CD8^+^ T cells. We also described how CD8^+^ T cells were coerced to exhaustion by Tregs, MDSCs, and TAMs after losing the assistance of DCs.

We summarized the crosstalk between immune cells-CD8^+^ Tex. Therefore, immunotherapy for tumor patients should not only focus on the anti-tumor immunity of tumor cells themselves, but also on the immunosuppressive cells in the TME that help tumor cells survive and escape. The immunosuppressive cells of the immune system in the body are not naturally the helper of tumor cells but interfere in the TME and stand on the opposite side of CD8^+^ T cells. However, it is not enough for our review to focus only on the crosstalk of CD8^+^ Tex with immune cells in the TME. Because complex TME also includes cancer-associated fibroblasts and other cells. How these cells together with immune cells create an immunosuppressive environment can be further explored. In addition, we also hope that the research of ICB therapy and ACT can also pay more attention to the crosstalk between immune cells-CD8^+^ Tex to create the possibility of improving effect. In conclusion, we believe that the immune system of healthy people is in a process of dynamic balance. The negative effects of immune cells in the TME need to be reversed in anti-tumor treatment, and new treatment methods should be found to make it become a roadblock for CD8^+^ T cells to become exhausted and a defender of anti-tumor immune effects.
